# Editorial for Special Issue: Advances in Smart and Tough Hydrogels

**DOI:** 10.3390/gels9100789

**Published:** 2023-10-01

**Authors:** Dong Zhang, Jintao Yang, Xiaoxia Le, Dianwen Song

**Affiliations:** 1 The Wallace H. Coulter Department of Biomedical Engineering, Georgia Institute of Technology and Emory University, Atlanta, GA 30332, USA; 2 College of Materials Science & Engineering, Zhejiang University of Technology, Hangzhou 310014, China; yangjt@zjut.edu.cn; 3 Ningbo Institute of Materials Technology and Engineering, Chinese Academy of Sciences, Ningbo 315201, China; lexiaoxia@nimte.ac.cn; 4 School of Medicine, Shanghai Jiaotong University, Shanghai 200240, China; dwsong@sjtu.edu.cn

## 1. Introduction

Smart hydrogels possess both intelligent and responsive properties, which are designed to exhibit specific responses to external stimuli such as changes in temperature, pH, or the presence of specific ions/counterions, making them “smart” or “responsive” materials. Tough hydrogels are engineered to have exceptional mechanical strength/toughness, which means they can endure significant mechanical stress or deformation without breaking or losing their structural integrity. Both of them have a wide range of potential applications in fields such as biomedicine, tissue engineering, drug delivery, and soft robotics, where their combination of responsiveness and mechanical resilience can be highly advantageous.

**Figure 1:**
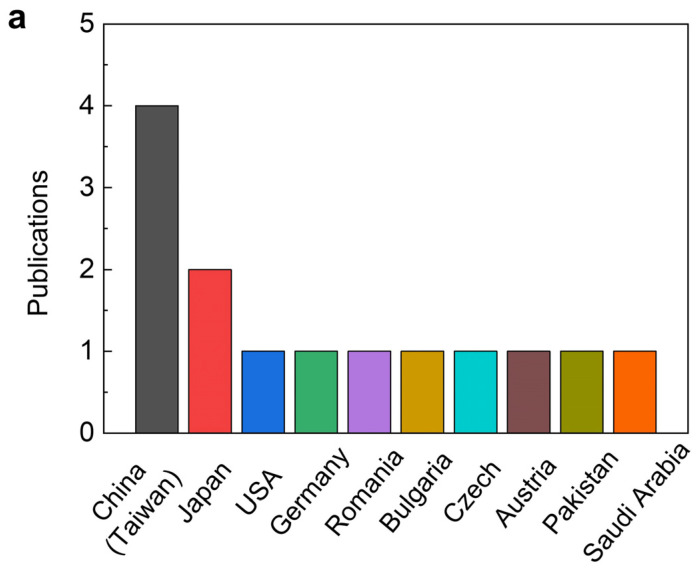

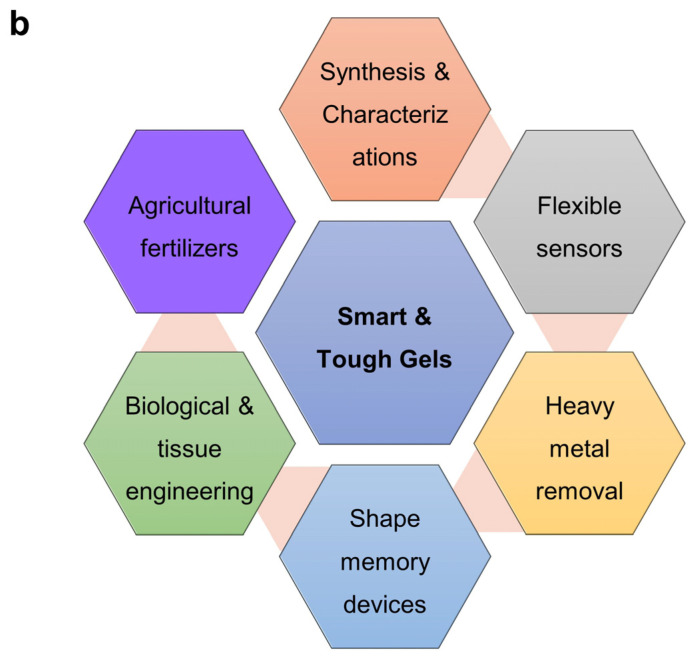
Summary of the *Advances in Smart and Tough Hydrogels* as a Special Issue of *Gels*. (**a**) Prominent contributing countries in research articles and (**b**) key themes in smart and tough hydrogels explored for this Special Issue.

This Special Issue features contributions from prominent experts in the field, with substantial input from 53 researchers representing more than 10 diverse regions worldwide (Figure 1a). These regions encompass countries such as China (including Taiwan), Japan, the USA, Germany, Romania, Bulgaria, the Czech Republic, Austria, Pakistan, and Saudi Arabia. Significantly, the important research collection included essential synthesis and characterization processes, along with practical applications spanning biological and tissue engineering, heavy metal removal, flexible sensors, shape memory devices, and agricultural fertilizers, among others (Figure 1b).

## 2. Contributions

It is evident that over an extended period, the quest for a comprehensive understanding of gelation phenomena, coupled with the discovery of innovative gelation mechanisms and systems, has remained a pressing and unmet need. This ongoing pursuit underscores the continuous demand for advancements in this field, driven by the ever-evolving challenges and opportunities it presents. With this mission, Yutaka et al. contributed an intriguing gelation phenomenon: the intelligent gelation occurring at low concentrations when combining the polymer hydrogelator NaPPDT with the water-soluble polymer poly(vinyl alcohol) (PVA) [1]. This unique behavior is exclusive to the NaPPDT and PVA combination, as it is not observed when mixing aqueous anionic polymer solutions with other sodium sulfonate or phosphonic acid side chains and aqueous PVA solutions. More interestingly, even when diluting the concentrations of the gelator and PVA in aqueous solutions, the composite gel materials exhibit improved mechanical properties.

Not all chemical gelation processes exhibit stability. Indeed, the potential for degradable dynamics holds great promise, particularly in the context of injectable hydrogels. The Weichold group conducted a comprehensive assessment of the durability of alkaline hydrogels utilizing a widely used crosslinker, N,N′-methylenebisacrylamide (MBAA), and three recently introduced tetraallyl crosslinkers [2]. Upon subjecting the hydrogel models to accelerated aging at 60 °C for 28 days, those crosslinked with MBAA eventually transitioned into a liquid state, whereas the storage modulus and degree of swelling of the hydrogels crosslinked with the tetraallyl compounds which remained unaltered. This work significantly addresses the current knowledge gap in our understanding of the long-term performance and durability of synthetic hydrogels when subjected to hydrolytic conditions, while it simultaneously provides valuable insights into the practical utility and longevity of these crosslinked systems. Structurally, Marin et al. developed a novel hydrogel based on poly(acrylic acid-*co*-acrylamide)/polyacrylamide pseudo-interpenetrating polymer networks (pIPNs), with the inclusion of magnetite achieved through the in situ precipitation of Fe(III)/Fe(II) ions within the hydrogel matrix [3]. This unique design conferred upon the hybrid hydrogels the ability to respond to pH and ionic strength variations, in addition to endowing them with superparamagnetic properties. This breakthrough highlights the potential of pIPNs as matrices for precisely controlling the deposition of inorganic particles, representing a promising approach for manufacturing structural soft matter. In essence, researchers are encouraged to utilize chemical tools/reactions for fine-tuning network distributions and structures in the development of smart and robust hydrogels, such as phase separation or macrophase separation strategies [4,5,6].

In the fields of smart medicines and biomedical tissue engineering, Wu and coworkers engineered a smart hydrogel by melding xanthan gum, Pluronic F-127, and synthetic monomer acrylic acid using the free radical polymerization technique [7]. Smart hydrogels exhibited minimal swelling behavior at pH 1.2 and 4.6, while they displayed increased drug release at pH 7.4, indicating their pH-responsive characteristics in regulating the release of atomoxetine HCl within the colon over an extended duration. The resulting hydrogels demonstrated an impressive level of intelligence and adaptability, underscoring their potential for diverse applications in drug delivery and control release. Zhang, Song, and colleagues embarked on the mission to tackle existing challenges in local fracture healing and early anti-osteoporosis therapy for osteoporosis [8]. Their approach involved designing injectable hydrogels loaded with calcium phosphate cement (CPC). The integration of this robust biomimetic hydrogel with bioactive CPC represents a highly promising and innovative contender for the development of commercial clinical materials aimed at improving the prognosis of patients grappling with osteoporotic fractures.

As for the applications of environmental science, Shi et al. demonstrated the remarkable adsorption capabilities of smart hydrogels using a functionalized sodium alginate hydrogel (FSAH) [9]. This hydrogel was specifically engineered for the efficient removal of heavy metals and dyes by incorporating hydrazide-functionalized sodium alginate with hydrazone groups for the selective capture of heavy metals (Pb^2+^, Cd^2+^, and Cu^2+^), while another dopamine grafting functional group offers active sites for adsorbing methylene blue, malachite green, and crystal violet. Notably, even after undergoing five adsorption–desorption cycles, it retained over 70% efficiency in the removal process. The encapsulation of hydrophobic molecular compounds into a polymer matrix has emerged as a method to modulate low solubility in water and a promising approach to preserve their chemical integrity, efficacy, but also their controlled release in a pulsating or continuous regime. A new cryogel system based on PVA and poly(ethylene brassylate-*co*-squaric acid) (PEBSA) obtained by repeated freeze–thaw processes, showing the cumulative antioxidant efficiency and antimicrobial activity against *E. coli* (Gram-negative strain), *S. aureus* (Gram-positive strain), and *C. albicans* (fungal strain) [10]. In addition, Martina’s group employed intelligent superabsorbent polymeric hydrogels, utilizing them as both a water reservoir and a supplier of mineral and organic nutrients with the aim of enhancing soil quality. In this case, the incorporation of NPK fertilizer reinforced the flexibility of the hydrogels, whereas the inclusion of lignohumate induced a shift in their rheological properties towards a more liquid-like behavior [11]. In light of the growing interest and attention toward hydrogel-based smart soils and relevant sustainable devices for tackling agricultural and environmental challenges, we recommend considering the following perspectives of smart hydrogels: (i) enhancing moisture retention and irrigation efficiency, (ii) implementing controlled nutrient release mechanisms, (iii) integrating soil health monitoring and environmental protection, (iv) ensuring compatibility with soil microorganisms, and (v) exploring additional avenues [12].

In hydrogel-based smart devices, Hideaki and colleagues unveiled that poly(stearyl acrylate (SA)-*co*-methoxy poly(ethylene glycol) acrylate (MPGA)) hydrogels featuring XSA > 0.5 trigger a transition from crystalline to amorphous state, constituting a challenging shift from hardness to softness at approximately 40 °C [13]. These hydrogels exhibited remarkable volume stability, irrespective of temperature fluctuations. This distinct attribute led to the utilization of poly(SA-*co*-MPGA) hydrogel in the development of shape memory “devices”, rendering them pliable and flexible at temperatures exceeding 40 °C, and stiffening when cooled below 37.5 °C. Hydrogels with a high water content (50~99%) are susceptible to freezing, leading to reduced flexibility at low temperatures, significantly restricting their utility in cold environments. To address this, Tang and coworkers employed butanediol (BD) and N-hydroxyethyl acrylamide (HEAA) monomers, both featuring a multi-hydrogen bond structure, to fabricate a LiCl/p(HEAA-*co*-BD) conductive hydrogel with anti-freezing properties [14]. They achieved this by strategically manipulating intermolecular and intramolecular hydrogel bonds within the crosslinking network, effectively inhibiting the formation of primary ice crystals. The designed smart hydrogel-based sensors enabled an excellent anti-freezing property with a low freeze point of −85.6 °C, while they maintained stretchability up to 400% with a tensile stress of ~450 kPa for human motion detection at −40 °C. Although smart hydrogel devices offer numerous advantages, including flexibility, biocompatibility, and sensitivity, they do have certain limitations, such as limited long-term stability, low gauge factor, and slow response time, among others. Continual research and innovations in this field are poised to tackle these hurdles, broadening the scope of potential applications, and enhancing the overall efficacy of hydrogel devices across diverse domains such as healthcare, environmental monitoring, and beyond.

## 3. Conclusions

We aspire for this Special Issue to offer readers insightful glimpses into the synthesis, characterization, and applications of both smart and tough hydrogels. Given the dynamic and rapidly advancing nature of this field, it is unfeasible to encompass every facet, particularly the recent breakthroughs from research groups not directly involved in this Special Issue. Undoubtedly, this burgeoning field will continue to flourish, drawing contributions from diverse disciplines such as chemistry, physics, materials science, and engineering. We also hope that readers will derive both enjoyment and inspiration from the diverse array of topics presented here, potentially propelling this field closer to the commercial significance of soft hydrogels in various promising fields.
